# Association Between Industrialized Dietary Pattern and Cardiometabolic Risk Factors in Children With FTO RS9939609 Gene Polymorphism

**DOI:** 10.1002/mnfr.70254

**Published:** 2025-09-04

**Authors:** Bhreendda’ Hary Dy Luar Prates Kiepper, Francilene Maria Azevedo, Aline Carare Candido, Mariane Alves Silva, Juliana Farias de Novaes, Cristina Maria Mendes Resende, Danielle Fernandes Durso, Jacqueline Isaura Alvarez‐Leite, Gustavo Velasquez‐Melendez, Sylvia do Carmo Castro Franceschini, Eliana Carla Gomes de Souza, Sarah Aparecida Vieira Ribeiro

**Affiliations:** ^1^ Department of Nutrition and Health Postgraduate Program in Nutrition Science Federal University of Viçosa Viçosa Brazil; ^2^ Graduate Program in Hebiatria University of Pernambuco Recife Brazil; ^3^ Department of Biochemistry and Immunology Federal University of Minas Gerais Belo Horizonte Brazil; ^4^ School of Nursing Federal University of Minas Gerais Belo Horizonte Brazil

**Keywords:** cardiometabolic risk factors, child, dietary pattern, polymorphism single nucleotide

## Abstract

Dietary patterns may increase cardiometabolic risk, especially in genetically predisposed individuals. Thus, the present study evaluated the association between dietary patterns and cardiometabolic risk factors in children with fat mass and obesity associated (FTO) gene polymorphism. A cross‐sectional survey of 258 children aged 4–7 years. Body composition was determined with dual‐energy x‐ray absorptiometry (DEXA). Biochemical samples and blood pressure were analyzed. Genotyping of rs9939609 was performed using oral swab samples and the TaqMan SNP test. Multiple linear regression stratified by FTO gene categories analyzed the association between dietary patterns and cardiometabolic risk factors. The prevalence of polymorphism was 20.2%. Five dietary patterns were identified: “Traditional”, “Industrialized”, “Milk and chocolate milk”, “Snack”, and “Natural”. Associations were found between the “Industrialized” dietary pattern and both the triglyceride‐glucose (TyG) index (*β* = 0.06; 95% confidence interval [CI]: 0.01–0.11) and triglycerides (*β* = 7.47; 95% CI: 0.73–14.21) in children with polymorphism. Additionally, “Milk and Chocolate Milk” pattern was associated with the TyG index (*β* = 0.03; 95% CI: 0.00–0.07) in children with a risk allele. For children with FTO gene polymorphism, adherence to the “Industrialized” dietary pattern was associated with cardiometabolic risk, highlighting the need for nutritional strategies to prevent.

## Graphical abstract

This study examined how dietary patterns affect health in children aged 4–7 years, especially in the presence of the fat mass and obesity associated (FTO) gene polymorphism. Researchers found that children with FTO gene polymorphism who ate more industrialized foods had higher risks of health problems and cardiometabolic risk with higher values of the triglyceride‐glucose (TyG) index and triglycerides.

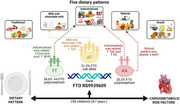

**Abbreviations**
AA: presence of polymorphism
AT: presence of risk allele
BF%: body fat
BMI: body mass index
CI: confidence interval
DBP: diastolic blood pressure
DEXA: dual‐energy x‐ray absorptiometry
EBF: exclusive breastfeeding
FP: fat percentage
FTO: fat mass and obesity associated
MAP: mean arterial pressure
PROLAC: Lactation Support Program
SBP: systolic blood pressure
TC: total cholesterol
TG: triglyceride
TT: no polymorphism
TyG: triglyceride‐glucose
WHR: waist‐to‐height ratio

## 1 Introduction

Excess weight has become increasingly prevalent in children. In the last four decades, its prevalence has increased more than 10‐fold [1]. Today, overweight and obesity are considered a global epidemic and are expected to affect more than 70 million children in 2025 [2]. This projection is concerning since overweight children are more likely to develop cardiometabolic comorbidities into adulthood [3, 4, 5], including dyslipidemia, arterial hypertension, and metabolic syndrome [5, 6, 7].

Numerous studies on children have found a relationship between diet and noncommunicable diseases [8, 9]. A greater tendency for nutrition inadequacies in childhood is believed to be related to the reduced and increased consumption, respectively, of fresh foods and processed foods [10, 11, 12]. Inadequate dietary patterns often reflect broader environmental influences, including home, school, and community contexts [13, 14, 15].

Thus, assessing food consumption according to dietary patterns is an important approach to investigate diet from a global perspective. Besides, it facilitates the development of strategies aimed at healthy eating and the prevention of diseases and nutrition problems [11, 12, 16, 17]. Concomitant with inadequate eating habits and increased cardiometabolic risk, there are implications of genetics in this relationship during childhood [18, 19]. The presence of the fat mass and obesity associated (FTO) gene in the DNA cascade predisposes one to excess weight and changes in satiety, increasing the risk of developing obesity and associated diseases, especially when structural variations occur in the nucleotide chains, triggering polymorphisms that directly affect the human genome [20, 21, 22, 23].

The FTO gene polymorphism is related to energy homeostasis and appetite regulation, which reinforces the importance of its identification since childhood, a period of establishment of eating behaviors that can influence health throughout life [21, 22]. Moreover, early childhood (Ages 4–7) is a sensitive developmental window marked by rapid growth, metabolic programming, and behavioral plasticity, a period which nutritional and genetic interactions can shape long‐term metabolic health. Genetic polymorphisms can alter the metabolic response to nutrients, modulating predispositions to obesity and metabolic dysfunctions. This approach allows early and personalized interventions, aiming at the prevention of adverse cardiometabolic outcomes. Despite this fact, few studies have evaluated the relationship between eating habits and cardiometabolic risk in children with genetic polymorphism [19, 20, 21, 22, 23].

Moraes et al. [21] observed that carriers of the FTO polymorphism exhibited distinct cardiometabolic profiles and responses following dietary interventions. Supporting this, Petermann et al. [19] demonstrated that this polymorphism is linked to an elevated risk of developing obesity‐related conditions in children, suggesting that genetic susceptibility may amplify the adverse effects of unhealthy dietary habits. Similarly, Pereira‐Filho et al. [23] reinforced the relevance of FTO and their significant impact on obesity and related metabolic disorders, which are often exacerbated by poor dietary patterns.

Thus, the present study aimed to evaluate the relationship between dietary pattern and cardiometabolic risk in children aged 4–7 years carrying the FTO gene polymorphism. We hypothesize that unhealthy eating patterns are associated with a greater predisposition to cardiometabolic risk among genetically susceptible children in this age group.

## 2 Materials and Methods

### 2.1 Study Design and Sample

The study has a cross‐sectional design. It was conducted in Viçosa (Minas Gerais, Brazil) with children aged 4–7 years who were monitored in their first year of life by the Lactation Support Program (PROLAC).

Information on the identification and location of the children was collected from PROLAC nutritional records. The inclusion criteria consisted of the presence of identification data that allows the location of the child and date of birth corresponding to ages between 4 and 7 years at the time of the study. A total of 669 children were eligible for the study; however, 176 were not located, 75 were not authorized by parents to participate or did not complete all stages of the study, and 8 had health problems related to their nutritional status, body composition, lipid profile, blood pressure, and glucose metabolism that disallowed participation. In addition, 7 children were excluded for having incomplete food consumption data. Thus, 403 children participated in the first stage of the process, 266 children (39.8%) being considered as sample loss.

In the second stage, information regarding home address and school details was updated via phone calls to parents and guardians. Subsequently, the principals of the respective schools in Viçosa were contacted to collect the genetic materials (swab method) of the included children. Thus, the number of children whose genetic material was collected was 258.

A power analysis was conducted in the OpenEpi online program, considering the prevalence of overweight in children with FTO gene polymorphism (78.6%), without polymorphism (21.4%), and a significance level of 95%. Based on these considerations, the power of the study was 100%.

This work is part of two larger studies entitled “Genetic and environmental determinants, dietary intake, and weight and body composition according to two cohorts of children from birth to 7 years old” and “dietary pattern, body adiposity and cardiometabolic risk factors in children from 4 to 7 years of age”, both submitted and approved by the Ethics Committee for Research with Human Beings of the Federal University of Viçosa (no. 663.171/2014; no. 892476/2014).

### 2.2 Nutritional Status and Cardiometabolic Risk Factors

Anthropometric data such as weight, height, and waist circumference were obtained according to recommended techniques [24]. Weight was measured on an electronic digital scale with a capacity of 150 kg and 10 g precision. Height was measured with a 2‐m wall‐mounted stadiometer, divided into centimeters and subdivided into millimeters.

Body mass index (BMI) for age (BMI/A) was calculated according to sex, as recommended by the World Health Organization [25]. Waist circumference was measured at the level of the umbilical scar using a 2‐m flexible and inelastic measuring tape, divided into centimeters and subdivided into millimeters [26].

The measurements were done in triplicate, and the mean was estimated from the two closest values. Waist‐to‐height ratio (WHR) was calculated as the ratio of waist circumference (cm) to height (cm), and values > 0.5 [27] were classified as cardiometabolic risk.

Biochemical tests were performed at the Clinical Analysis Laboratory, Health Division, Federal University of Viçosa. After 12 h fasting, blood samples of the children were collected by venipuncture. Blood glucose and lipid profile markers were measured (total cholesterol [TC], HDL‐c, LDL‐c, and triglycerides [TGs]). Based on these markers, cholesterol (non‐HDLC‐c) and triglyceride‐glucose (TyG) index were calculated. The TyG index is an indirect measure of insulin resistance, calculated as: ln [fasting TGs (mg/dL) × fasting glucose (mg/dL)]/2 [28]. Dyslipidemia was classified in the children as having alterations in at least one of these parameters: TC ≥ 170 mg/dL, HDL‐c < 45 mg/dL, LDL‐c ≥110 mg/dL, and TG ≥75 mg/dL [29].

Using an automatic insufflation blood pressure monitor (Omron Model HEM‐741 CINT) recommended by the Brazilian Society of Cardiology [30], blood pressure was measured following the protocols outlined by the VII Brazilian Directive on Hypertension. Three measurements were performed with intervals of at least 1 min, and the mean of the last two measurements was considered. For the analyses, the mean between the systolic blood pressure (SBP) and diastolic blood pressure (DBP) was used, resulting in mean arterial pressure (MAP) calculated as: MAP = [DBP + 1/3 (SBP − DBP)] [31].

### 2.3 Assessment of Dietary Patterns

The food consumption of the children was assessed through three food records completed by the children's guardians/patients, on non‐consecutive days and on a weekend (1 record). The records were checked and revised by the researchers to reduce reporting bias, and dietary data were entered and processed using Dietpro software, version 5i.

Dietary pattern analysis was performed by grouping the reported foods and preparations according to nutritional characteristics or botanical composition, resulting in 14 predefined food groups [32].

To identify dietary patterns, a posteriori approach was used. Principal component analysis (PCA) was performed based on the correlation matrix of food groups. Sampling adequacy was confirmed using the Kaiser–Meyer–Olkin (KMO) index (KMO = 0.521) and Bartlett's test of sphericity (*p* < 0.001). A varimax orthogonal rotation was applied to improve factor interpretability, and the number of components retained was determined through visual inspection of the scree plot (Cattell's criterion).

Foods/groups with factor loads ± > 0.3 were considered to contribute meaningfully to the component structure, facilitating the labeling of dietary patterns. The naming of the patterns was based on the dominant food groups in each component, following nutritional and botanical similarities, and consistent with terminology used in previous studies [12, 32, 33, 34].

After identifying the components, individual factor scores were computed for each participant by applying the weighted sum of standardized food group intakes, ensuring comparability across individuals. These scores represent the degree of adherence to each dietary pattern.

Missing dietary data was handled using complete case analysis. Participants with incomplete dietary records were excluded from the PCA to avoid imputation bias.

### 2.4 Genotyping

Oral swab was utilized for genetic material collection, repeating the process twice. The brushes were kept in cold ethanol until DNA extraction. For DNA extraction, the SYBR Green Extract ‐N‐ Amp Tissue Kit (Sigma) was used. The polymerase chain methodology (TaqMan SNP Genotyping Assays) was used in real time (PCR) to discriminate the alleles, typifying the genetic sample for the SNP studied. The SNP Taq‐ManTM genotyping methodology was based on predesigned and validated assays produced by Applied Biosystems (ABI, catalog number: 4351379).

In all repetition plates, the same genetic material plus a positive control was used to identify any typos and white reaction controls, which contain autoclaved double‐distilled water instead of DNA and named by the no template control (NTC) programs.

The programs used for the plate design and result visualization were SDS v2 (Applied Biosystems), which is composed of four softwares and a TaqManTM genotyping software, respectively. The TaqManTM SNP genotyping methodology of the specific ABI utilized for the FTO locus study is commercially classified as C_30090620_10.

### 2.5 Independent Variables

Data on duration of exclusive breastfeeding (EBF) and birth weight were obtained from PROLAC nutritional records. In relation to data about the children during the periods between 4 and 7 years, the parents/guardians completed a semi‐structured questionnaire on sociodemographic information, namely: maternal education and income per capita, both categorized in tertiles.

Information on lifestyle habits was obtained through a questionnaire adapted from Andaki [35]. The variable evaluated was daily screen time (television, computer, and games), where a daily screen time of more than 2 h is a risk factor, according to the American Academy of Pediatrics [36]. Parents were asked about the child's history of familial dyslipidemia (father or mother), and this variable was categorized into “presence” or “absence”.

### 2.6 Data Analysis

The distribution of variables was assessed using the Shapiro–Wilk normality test. Descriptive analysis was presented in measures of frequency distribution, mean, and standard deviation. For comparison of means, Student's *t* test or ANOVA (with Bonferroni post hoc) tests were used.

To assess the association between dietary patterns (explanatory variable) and cardiometabolic risk factors (outcomes), multiple linear regression was applied and adjusted for potential confounders like EBF duration, family history of dyslipidemia sociodemographic variables (age, sex, socioeconomic status), lifestyle (screen time), and body composition (BMI and fat percentage [FP]). The adjustment variables were selected based on the literature, and regression analysis was stratified according to FTO gene categories (no polymorphism [TT], presence of risk allele [AT], and presence of polymorphism [AA]). This model was chosen because the dependent variables were used in their continuous nature.

To verify the adequacy and adjustment of the linear regression model, the normality of residuals and heteroscedasticity were evaluated through Q–Q plots and Shapiro–Wilk test. Residuals were considered normally distributed if they followed the theoretical quantile line in the plot and the normality test yielded *p* >0.05. The analyses were performed using SPSS software version 21.0 and Stata software version 13.0. The statistical significance of *α* = 5% was respected.

The chi‐square test of Hardy–Weinberg equilibrium was conducted to verify equilibrium in the FTO genotypic frequencies (rs9939609) presented in the study population, and the *p* value was higher than 0.05, which indicates no population stratification bias.

## 3 Result

A total of 258 children participated in this study, with a mean age of 5.9 ± 1.0 years; 52.7% were boys, 74% had dyslipidemia, and 75.8% had a history of familial dyslipidemia. The prevalence of a risk allele was 51.2% and that of the FTO gene polymorphism was 20.2%. Furthermore, the most clinically relevant finding was that children with the FTO polymorphism who adhered to the “Industrialized” dietary pattern showed higher TyG index and TGs.

A higher mean TyG index was found in children who did not receive EBF in the first 4 months of life, daily screen time > 2 h, and presence of history of familial dyslipidemia. Mean TG, LDL, cholesterol (non‐HDL), and TC were higher in children with a family history of dyslipidemia. Also, mothers with a higher level of education had higher TC. Children who had higher BMI and FP presented higher MAP, WHR, and blood glucose. Male and older children had higher mean blood glucose levels. Mean HDL cholesterol was lower among the younger children (Table S1).

There were no significant differences in mean cardiometabolic risk factors between the FTO gene categories. However, 53.1% of the children with at least one risk allele (AT) presented a cardiometabolic risk marker, and 50.0% had two or more than three markers (Table S2).

Five dietary patterns were identified, which explained 54.6% of the data variance (Table 1). The first dietary pattern was called “Traditional”, as it presents foods/preparations characteristic of the Brazilian diet, such as: beans; vegetables and legumes; rice, tubers, and cornmeal mush; meat and eggs. The second standard was designated “Industrialized”, as it mainly contains foods/groups rich in fat and sugar, such as: stuffed biscuits and sweets; artificial juices and soft drinks; fried foods, snacks, and sausages. The pattern “Milk and chocolate milk” exclusively represents milk and chocolate milk, due to their high intake among children in the study. The characteristic “Snack” pattern was represented by foods such as: breads, cereals, cakes, and biscuits; butter and margarine; and coffee. The fifth pattern observed was named “Natural”, as it mainly represents: natural juice and fruits; broths, soups, and pasta.

**TABLE 1:** Dietary patterns and factor loadings of food groups consumed by children, Viçosa, Minas Gerais, Brazil, 2015–2016.

Food/groups	Dietary patterns				
	Traditional	Industrialized	Milk with chocolate milk	Snack	Natural
Milk and derivatives	−0.074	−0.188	**0.767**	0.164	0.082
Chocolate milk with sugar	−0.076	0.027	**0.852**	−0.007	−0.039
Coffee	−0.142	**−0.322**	**−0.422**	**0.435**	−0.007
Butter and magarine	0.254	0.037	0.057	**0.605**	−0.059
Breads, cereals, cakes, and biscuits	−0.062	−0.001	0.032	**0.823**	0.061
Stuffed biscuits and sweets	0.002	**0.536**	−0.054	−0.142	0.475
Beans	**0.624**	−0.172	−0.168	0.023	0.287
Vegetables and legumes	**0.567**	0.017	0.034	0.095	0.088
Artificial juices and soft drink	−0.150	**0.792**	−0.037	0.043	−0.146
Rice, tubers, and mush (cornmeal)	**0.762**	−0.163	−0.131	0.075	−0.134
Meat and eggs	**0.516**	0.192	0.132	−0.046	**−0.346**
Fried foods, snacks, and sausages	−0.004	**0.705**	−0.025	0.036	0.049
Natural juice and fruits	0.110	0.033	0.138	0.044	**0.780**
Broths, soups, and pasta	−0.164	−0.084	−0.180	**−0.349**	**0.347**
% Of explained variance	14.2	11.8	10.8	9.4	8.3
Total explained variance	54.6%				

*Note*: Extraction method: principal component analysis. Varimax rotation with Kaiser normalization. Values in bold represent factor loadings ±>0.3.

Among children with FTO polymorphism (AA), a positive association was observed between “Industrialized” dietary pattern and TyG index (*β* = 0.06; 95% confidence interval [CI]: 0.01–0.11). Similarly, for children with FTO polymorphism (AA), “Industrialized” pattern score correlates with increase in TGs (*β* = 7.47; 95%CI: 0.73–14.21). Among children without any risk allele (TT), there was a positive association between WHR and “Industrialized” dietary pattern (*β* = 0.01; 95% CI: 0.00–0.02), and a negative association between the same dietary pattern and blood glucose (*β* = −2.27; 95% CI: −4.45 to −0.08). In addition, a positive association was observed between the dietary pattern “Milk and chocolate Milk” and TyG index among children with a risk allele (AT) (*β* = 0.03; 95% CI: 0.0–0.07). Among children carrying the FTO gene polymorphism, higher consumption of natural foods such as fruits and fruit juices was significantly associated with lower blood pressure levels (*β* = −2.43; 95% CI: −4.87 to −0.01) (Table 2). Moreover, “Traditional” and “Snack” dietary patterns were unassociated with cardiometabolic risk factors (Table S3).

**TABLE 2:** Association between dietary patterns (explanatory variables) and cardiometabolic risk factors (dependent variables) in children according to FTO genotype, Viçosa, Minas Gerais, Brazil, 2015–2016.

	Industrialized			Milk and chocolate milk			Natural		
Variables	TT *β* (CI95%)	AT *β* (CI95%)	AA *β* (CI95%)	TT *β* (CI95%)	AT *β* (CI95%)	AA *β* (CI95%)	TT *β* (CI95%)	AT *β* (CI95%)	AA *β* (CI95%)
TyG^a^	−0.05 (−0.11–0.01)	0.0 (−0.04–0.03)	0.06 (0.01–0.11)^*^	−0.02 (−0.07–0.03)	0.03 (0.0–0.07)^*^	−0.01 (−0.07–0.06)	−0.02 (−0.07–0.02)	0.0 (−0.03–0.04)	0.01 (−0.06–0.08)
MAP^a^	−0.05 (−2.40–2.29)	−0.26 (−1.69–1.16)	−0.32 (−2.12–1.49)	−0.21 (−2.10–1.68)	0.39 (−1.02–1.80)	−0.72 (−2.92–1.48)	−0.52 (−2.29–1.24)	−0.52 (−2.01–0.93)	−2.43 (−4.87–0.01)^*^
WHR^b^	0.01 (0.00–0.02)^*^	0.0 (−0.02–0.02)	0.0 (−0.01–0.01)	0.01 (0.0–0.01)	0.0 (0.0–0.01)	0.0 (−0.02–0.01)	−0.01 (−0.01–0.0)	0.01 (−0.0–0.01)	−0.01 (−0.02–0.01)
TG^a^	−3.16 (−11.06–4.74)	−1.49 (−6.56–3.59)	7.47 (0.73–14.21)^*^	−0.99 (−7.29–5.29)	4.87 (−0.08–9.81)	−1.95 (−10.65–6.75)	−0.90 (−6.62–4.83)	−1.51 (−6.80–3.79)	−0.69 (−10.77–9.40)
LDL^a^	3.18 (−3.37–9.74)	−2.13 (−6.26–1.99)	−1.56 (−10.71–7.59)	2.25 (−2.96–7.45)	−0.74 (−4.84–3.36)	−3.77 (−14.92–7.37)	2.33 (−2.39–7.06)	2.30 (−2.01–6.61)	−4.65 (−17.52–8.23)
HDL^a^	0.72 (−2.99–4.44)	0.71 (−1.34–2.76)	2.12 (−0.52–4.75)	0.25 (−2.70–3.20)	−1.96 (−3.96–0.05)	0.61 (−2.71–3.92)	1.72 (−0.93–4.36)	−0.54 (−2.68–1.61)	−0.42 (−4.26–3.41)
Cholesterol (non‐HDL)^a^	2.55 (−4.29–9.40)	−2.46 (−6.83–1.91)	−0.12 (−9.73–9.48)	2.06 (−3.36–7.49)	0.26 (−4.06–4.63)	−4.16 (−15.83–7.52)	2.26 (−2.67–7.19)	1.95 (−2.63–6.53)	−4.68 (−18.19–8.83)
Total cholesterol^a^	3.28 (−4.14–10.69)	−1.75 (−6.23–2.73)	1.99 (−7.83–11.82)	2.31 (−3.57–8.19)	−1.67 (−6.11–2.77)	−3.55 (−15.54–8.44)	3.98 (−1.31–9.27)	1.41 (−3.28–6.10)	−5.11 (−18.93–8.73)
Blood glucose^a^	−2.27 (−4.45 to −0.08)^*^	0.73 (−0.27–1.73)	1.26 (−0.31–2.83)	−1.64 (−3.39–0.11)	0.26 (−0.74–1.26)	0.48 (−1.50–2.45)	−1.42 (−3.03–0.19)	0.50 (−0.55–1.56)	1.40 (−0.85–3.65)

*Note*: Linear regression. *p < 0.05.

Abbreviations: 95%CI, confidence interval (95%); AA, presence of polymorphism; AT, presence of a risk allele; HDL‐c, HDL‐cholesterol; LDL‐c, LDL‐cholesterol; MAP, mean arterial pressure; TG, triglyceride; TT, no polymorphism; TyG, triglyceride‐glucose index; WHR, waist‐to‐height ratio.

^a^ Adjusted for age, sex, fat percentage, screen time, and maternal education.

^b^ Adjusted for age, gender, screen time, and maternal education.

**FIGURE 1:**
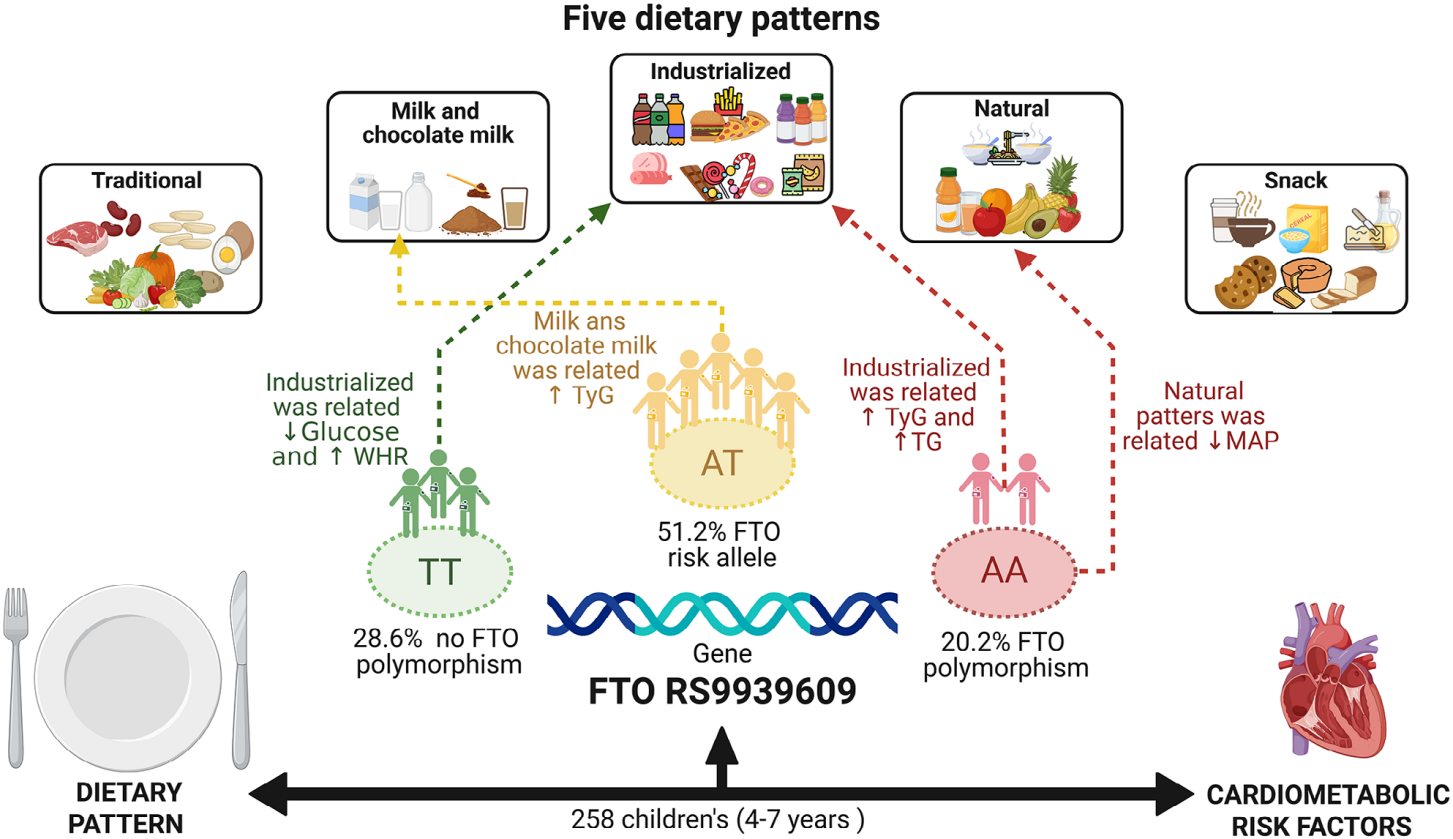
Association between dietary patterns (explanatory variables) and cardiometabolic risk factors (dependent variables) in children according to FTO genotype. Viçosa, Minas Gerais, Brazil, 2015‐2016. AA, presence of polymorphism; AT, presence of a risk allele; FTO, fat mass and obesity associated; MAP, mean arterial pressure; TG, triglyceride; TT, no polymorphism; TyG, triglyceride‐glucose index; WHR, waist‐to‐height ratio.

Figure 1 summarizes the foods that make up each identified dietary pattern and the observed relationships with cardiovascular risk factors for each group, specifically, children without any risk allele (TT), with a risk allele (AT), and with FTO polymorphism (AA).

## 4 Discussion

This study identified five dietary patterns (“Traditional”, “Industrialized”, “Milk and Chocolate Milk”, “Snack”, and “Natural”) among children aged 4–7 years. We found that greater adherence to the “Industrialized” dietary pattern was positively associated with TyG index and TGs in children with FTO gene polymorphisms, suggesting a gene–diet interaction that may increase the risk of early cardiometabolic disease. Furthermore, in children without a risk allele, the same pattern was negatively associated with blood glucose and positively associated with WHR, which are considered cardiometabolic risk factors. Thus, the combination of foods consumed in each dietary pattern reflects the children's genetic, cultural, social, environmental, economic, and health determinants [37]. And the differences between groups may reflect gene–environment interactions, where the AA influences metabolic responses to specific dietary patterns.

Regarding obesity, in our study, there was no association of the FTO gene with BMI and body fat (BF%). However, several studies have shown that the association between FTO gene polymorphism and obesity related outcomes may intensify with age. In Brazilian, Chinese, and Mexican children, the presence of the risk allele was linked to increased body mass, subcutaneous fat, and other adiposity indicators such as BMI, waist circumference, and lipid alterations [38, 39, 40]. These associations became more evident during adolescence, particularly after puberty and among females, suggesting that age and physiological changes may modulate the genetic influence on obesity.

In the present study, among children with the risk allele, higher consumption of processed foods (“Industrialized” pattern) was associated with higher TyG index and TG levels, important markers of insulin resistance. These results align with prior evidence that energy‐dense diets, especially rich in sugar and fats, contribute to metabolic dysfunction, particularly in genetically predisposed individuals. These metabolic changes interfere with the development, performance of daily activities and metabolic parameters in children [41, 42, 43]. Furthermore, the presence of FTO gene polymorphism leads to alterations in blood glucose and insulin, increase in BF%, as well as greater consumption of processed foods that corroborate cardiometabolic alterations [20, 44, 45].

Despite of Pereira et al. [46] found no association between the presence of FTO gene polymorphism with alterations in cardiometabolic risk markers in Brazilian children, the obese/overweight group subjects had higher BMI, higher fasting glucose, HOMA‐IR index, TC, low‐density lipoprotein, and TGs. On the other hand, Ranzenhofer et al. [47] found that even in nonobese children, variations of SNP in the FTO gene were found, being evidenced as age and consumption of calorie dense food increase.

Another result shown that in children with presence of at least one risk allele the TyG was associated with “Milk and Chocolate Milk” pattern. Similarly, Vilella et al. [48] conducted a cross‐sectional study of 1191 children (4–11 years old) of a cohort and observed an association between consumption of foods rich in carbohydrate, protein, and fats and the presence of at least one risk allele in the FTO gene, especially in overweight and obese children. Likewise, Cauchí et al. [49] observed that the AT increased the chances of being Type 2 diabetes, overweight, and obese into adulthood, when compared to those without the FTO gene.

On the other hand, we observed a tendency of negative association between “Natural” pattern and MAP (*p* = 0.051). In a study, children with healthier eating habits had lower blood pressure, as well as lower chances of developing cardiometabolic complications, such as hypertension, dyslipidemia, and hyperglycemia, associated with overweight and obesity [50]. Furthermore, Xi et al. [51] found that Chinese children with FTO gene polymorphism presented greater variations in blood pressure as well as BMI and were more likely to develop hypertension.

Another relevant finding of this study was changes in the rates of cardiometabolic risk markers. In studies conducted in Brazil, children with high alterations in TG and blood glucose and lower HDL levels were more likely to develop cardiometabolic risk [52]. This was also observed in children who experienced premature interruption of breastfeeding and those with excess weight. Thus, these factors contribute to a greater tendency to consume less healthy foods, leading to nutritional problems and predisposition to cardiometabolic risk [17].

These studies reinforce the complex interplay between genetics, diet, and demographic factors such as age, sex, and ethnicity, and support the relevance of our findings in a Brazilian pediatric sample. It is recommended to use multiple cardiometabolic risk indicators to assess children's nutritional status, and c are essential to understand the effects of genetic polymorphisms on these outcomes.Trevisano et al. [53], in a systematic review, emphasized that the influence of the FTO rs9939609 polymorphism on body composition can vary across life, becoming more pronounced in older populations. This underscores the need for longitudinal studies about impact of genetic susceptibility.

Similarly, Hardy et al. [54], found associations between the FTO gene variant and the development of metabolic syndrome. Their findings highlight not only the genetic contribution to metabolic risk but also how this relationship may differ by ethnic background and evolve across different life stages. These studies reinforce the notion that longitudinal studies are crucial to establish causal relationships, assess long‐term health implications, and develop timely, tailored interventions.

The new perspective provided by our study demonstrated that the same dietary exposure can cause different cardiometabolic outcomes depending on a child's genetic profile. This highlights the importance of appropriate nutritional strategies based on genetic profile, especially early in life, when preventive interventions are most effective.

The limitations of this study were the subjectivity of dietary pattern analysis, such as the criteria used in the food grouping and the number of factors retained. However, to minimize biases in the analyses, the criteria adopted at all stages were described in detail and based on the literature. As the strength, the gene‐stratified analysis and use of different markers of cardiometabolic risk are highlighted, and the assessment of children's habitual food consumption through three food records, which reduces individual variability of intake. Given that there are few studies with this age group, our findings can be useful to the clinical and scientific community and provide valuable insights to longitudinal research.

## 5 Conclusion

The findings of this study highlight that the adherence to “Industrialized” dietary pattern in children aged 4–7 years was directly associated with cardiometabolic risk, represented by TyG index and TGs, in the presence of FTO gene polymorphism. Thus, it reinforces the potential interaction between diet and genetic profile and its early implications for health.

Considering this, it is recommended that the implementation of early childhood nutrition policies that reduce access to processed foods and promote the consumption of fresh and minimally processed items. Furthermore, integrating genetic screening into public health programs may help identify vulnerable children and support the development of personalized nutritional strategies. Future longitudinal studies are essential to establish causal pathways between early dietary exposure, genetic predisposition, and adult cardiometabolic outcomes.

## Supporting information




**Supporting File 1**: mnfr70254‐sup‐0001‐SuppMat.docx.

## Data Availability

The data that support the findings of this study are available from the corresponding author upon reasonable request.
