# Supplementary material for: Association Between Industrialized Dietary Pattern and Cardiometabolic Risk Factors in Children With FTO RS9939609 Gene Polymorphism
Source: Mol Nutr Food Res. 2025 Sep 4;69(22):e70254. doi: 10.1002/mnfr.70254 (PMC12643185; doi:10.1002/mnfr.70254)
Supplement: Supplementary file 1 — Supporting File 1: mnfr70254‐sup‐0001‐SuppMat.docx. [file MNFR-69-e70254-s001.docx]

**Supplementary Table 1:** Distribution of cardiometabolic risk markers according to the sociodemographic and anthropometric characteristics, body composition and lifestyle of children aged 4 to 7 years. Viçosa, Minas Gerais, Brazil, 2015-2016.

| Characteristics | n | TyG | | MAP | | WHR | | TG | | LDL-c | |
| --- | --- | --- | --- | --- | --- | --- | --- | --- | --- | --- | --- |
|  |  | Mean (SD) | *p* value | Mean (SD) | *p* value | Mean (SD) | *p* value | Mean (SD) | *p* value | Mean (SD) | *p* value |
| Sex |  |  | 0,351 |  | 0,440 |  | 0,190 |  | 0,413 |  | 0,843 |
| Male | 136 | 4,26 (0,21) |  | 71,92 (8,79) |  | 0,48 (0,04) |  | 66,42 (28,53) |  | 97,55 (25,27) |  |
| Female | 122 | 4,25 (0,19) |  | 71,78 (6,48) |  | 0,47 (0,04) |  | 65,68 (25,66) |  | 100,61 (26,37) |  |
| Age |  |  | 0,922 |  | 0,023 |  | **<0,001 ^b^** |  | 0,077 |  | 0,732 |
| 4-5 | 115 | 4,24 (0,18) |  | 70,77 (7,21) |  | 0,48 (0,04) |  | 63,39 (22,98) |  | 100,05 (22,81) |  |
| 6-7 | 143 | 4,27 (0,21) |  | 72,72 (8,11) |  | 0,47 (0,04) |  | 68,22 (30,01) |  | 98,15 (25,64) |  |
| BMI |  |  | 0,091 |  | **< 0,001 ^b^** |  | **<0,001 ^b^** |  | 0,266 |  | 0,913 |
| Normal | 201 | 4,25 (0,20) |  | 70,74 (6,70) |  | 0,46 (0,03) |  | 65,51 (26,97) |  | 100,10 (24,61) |  |
| Overweight | 57 | 4,29 (0,19) |  | 75,74 (9,81) |  | 0,52 (0,06) |  | 68,05 (27,99) |  | 95,11 (23,40) |  |
| BF% |  |  | 0,200 |  | **< 0,001 ^b^** |  | **< 0,001 ^b^** |  | 0,400 |  | 0,600 |
|  | 88 | 4,23 (0,21) |  | 70,07 (7,07) |  | 0,45 (0,03) |  | 63,40 (27,14) |  | 100,98 (27,40) |  |
| High | 85 | 4,28 (0,19) |  | 74,30 (8,90) |  | 0,51 (0,04) |  | 66,46 (26,17) |  | 97,73 (21,11) |  |
| Birth weight |  |  | 0,180 |  | 0,323 |  | 0,145 |  |  |  | 0,362 |
| < 3000g | 84 | 4,24 (0,16) |  | 71,54 (6,99) |  | 0,47 (0,04) |  | 62,43 (21,25) | 0,067 | 98,23 (24,11) |  |
| ≥ 3000g | 174 | 4,27 (0,21) |  | 72,01 (8,13) |  | 0,48 (0,04) |  | 67,83 (29,49) |  | 99,37 (25,08) |  |
| EBF up to 4 months |  |  | **< 0,001 ^b^** |  | 0,400 |  | 0,100 |  | 0,400 |  | 0,800 |
| No | 155 | 4,26 (0,20) |  | 71,92 (8,79) |  | 0,48 (0,04) |  | 66,42 (28,53) |  | 97,55 (25,27) |  |
| Yes | 97 | 4,25 (0,19) |  | 71,78 (6,48) |  | 0,47 (0,04) |  | 65,68 (25,66) |  | 100,62 (23,37) |  |
| Screen time |  |  | **0,049 ^a^** |  | 0,200 |  | 0,300 |  | 0,900 |  | 0,600 |
| < 2h | 36 | 4,21 (0,19) |  | 72,65 (7,41) |  | 0,48 (0,04) |  | 59,56 (23,50) |  | 97,54 (21,20) |  |
| ≥ 2h | 220 | 4,27 (0,20) |  | 71, 70 (7,84) |  | 0,47 (0,03) |  | 67,04 (27,64) |  | 99,22 (24,98) |  |
| Familial dyslipidemia |  |  | **< 0,001 ^b^** |  | 0,516 |  | 0,326 |  | **0,002 ^a^** |  | **0,007 ^a^** |
| Yes | 53 | 4,34 (0,18) |  | 71,30 (6,61) |  | 0,48 (0,04) |  | 76,09 (28,13) |  | 107,59 (27,24) |  |
| No | 165 | 4,24 (0,20) |  | 71,34 (6,71) |  | 0,47 (0,04) |  | 63,61 (26,77) |  | 98,28 (22,63) |  |
| Maternal education |  |  | 2,540 |  | 1,010 |  | 0,136 |  | 0,358 |  | 0,169 |
| < 8 years anos | 95 | 4,24 (0,23) |  | 72,59 (8,93) |  | 0,47 (0,04) |  | 64,85 (30,86) |  | 95,56 (22,20) |  |
| 8 – 11 years anos | 126 | 4,26 (0,18) |  | 71,68 (6,68) |  | 0,48 (0,04) |  | 65,63 (24,88) |  | 100,63 (24,60) |  |
| > 11 years anos | 35 | 4,32 (0,16) |  | 70,46 (7,84) |  | 0,48 (0,04) |  | 72,31 (24,22) |  | 103,37 (28,70) |  |
| Per capita income |  |  | 0,396 |  | 0,086 |  | 0,849 |  | 0,509 |  | 0,939 |
| < 255,00 | 85 | 4,23 (0,19) |  | 72,35 (5,87) |  | 0,48 (0,04) |  | 62,81 (24,33) |  | 99,85 (24,79) |  |
| 255 - 475,17 | 85 | 4,27 (0,19) |  | 72,26 (6,99) |  | 0,47 (0,04) |  | 66,69 (27,74) |  | 99,27 (24,19) |  |
| > 475,17 | 85 | 4,27 (0,21) |  | 70,29 (7,50) |  | 0,47 (0,04) |  | 67,12 (27,63) |  | 98,53 (24,54) |  |

**Table 1:** *(Continued)*

| Characteristics | N | HDL-c | | Cholesterol (non-HDL) | | Total cholesterol | | Blood glucose | |
| --- | --- | --- | --- | --- | --- | --- | --- | --- | --- |
|  |  | Mean (SD) | *p* value | Mean (SD) | *p* value | Mean (SD) | *p* value | Mean (SD) | *p* value |
| Sex |  |  | 0,824 |  | 0,820 |  | 0,897 |  | **0,031 ^a^** |
| Male | 136 | 49,74 (11,42) |  | 110,83 (26,27) |  | 160,57 (27,59) |  | 82,8 (6,78) |  |
| Female | 122 | 51,07 (11,52) |  | 113,76 (24,84) |  | 164,84 (26,35) |  | 81,10 ± (5,72) |  |
| Age |  |  | **0,026 ^a^** |  | 0,617 |  | 0,295 |  | **0,011 ^a^** |
| 4-5 | 115 | 48,83 (10,11) |  | 112,75 (24,03) |  | 161,57 (26,24) |  | 80,98 (7,08) |  |
| 6-7 | 143 | 51,61 (12,35) |  | 111,79 (26,87) |  | 163,40 (27,74) |  | 82,80 (5,59) |  |
| BMI |  |  | 0,642 |  | 0,878 |  | 0,896 |  | **< 0,001 ^b^** |
| Normal | 201 | 50,51 (11,47) |  | 113,21 (25,81) |  | 163,72 (27,94) |  | 81,37 (6,29) |  |
| Overweight | 57 | 49,88 (11,55) |  | 108,72 (24,77) |  | 158,60 (23,41) |  | 84,20 (6,08) |  |
| BF% |  |  | 0,500 |  | 0,700 |  | 0,900 |  | **< 0,001 ^b^** |
| Normal | 88 | 49,5 (10,23) |  | 113,70 (28,35) |  | 163,16 (30,37) |  | 81,15 (7,34) |  |
| High | 85 | 51,28 (14,21) |  | 110,92 (22,11) |  | 162,2 (23,60) |  | 83,35 (6,05) |  |
| Birth weight |  |  | 0,784 |  | 0,273 |  | 0,407 |  | 0,547 |
| < 3000g | 84 | 51,18 (11,83) |  | 110,83 (25,98) |  | 162,01 (27,62) |  | 82,06 (5,20) |  |
| ≥ 3000g | 174 | 49,98 (11,30) |  | 112,89 (25,46) |  | 162,86 (26,84) |  | 81,96 (6,85) |  |
| EBF up to 4 months |  |  | 0,800 |  | 0,800 |  | 0,800 |  | **< 0,001 ^b^** |
| No | 155 | 49,74 (11,42) |  | 110,83 (26,27) |  | 160,56 (27,59) |  | 82,8 (6,78) |  |
| Yes | 97 | 51,07 (11,52) |  | 113,76 (24,84) |  | 164,84 (26,35) |  | 81,10 (5,72) |  |
| Screen time |  |  | 0,021 |  | 0,700 |  | 0,900 |  | 0,700 |
| < 2h | 36 | 46,81 (9,11) |  | 109,44 (22,30) |  | 156,25 (20,40) |  | 81,28 (5,62) |  |
| ≥ 2h | 220 | 51,00 (11,72) |  | 112,63 (26,16) |  | 163,62 (27,94) |  | 82,07 (6,45) |  |
| Familial dyslipidemia |  |  | 0,432 |  | **0,002 ^a^** |  | **0,003 ^a^** |  | 0,262 |
| Yes | 53 | 50,02 (9,70) |  | 122,81 (28,64) |  | 172,83 (30,46) |  | 82,25 (5,87) |  |
| No | 165 | 50,30 (11,11) |  | 111,08 (23,84) |  | 161,39 (24,63) |  | 81,62 (6,26) |  |
| Maternal education |  |  | 0,420 |  | 0,120 |  | **0,044 ^a^** |  | 0,426 |
| < 8 years | 95 | 49,42 (10,65) |  | 108,53 (23,41) |  | 157,95 (25,18) |  | 81,35 (6,43) |  |
| 8 – 11 years | 126 | 50,44 (12,46) |  | 113,68 (25,47) |  | 164,12 (26,52) |  | 82,27 (6,49) |  |
| > 11 years | 35 | 52,37 (9,04) |  | 118,11 (30,61) |  | 170,49 (31,41) |  | 82,74 (5,59) |  |
| Per capita income |  |  | 0,483 |  | 0,990 |  | 0,921 |  | 0,326 |
| < 255,00 | 85 | 49,94 (11,64) |  | 112,31 (26,11) |  | 162,25 (26,79) |  | 81,14 (6,90) |  |
| 255-475,17 | 85 | 49,87 (11,25) |  | 112,61 (25,31) |  | 162,48 (26,38) |  | 82,49 (5,88) |  |
| > 475,17 | 85 | 51,74 (11,46) |  | 112,07 (26,02) |  | 163,81 (28,27) |  | 82,31 (6,31) |  |

TyG: triglyceride-glucose index; MAP: mean arterial pressure; WHR: waist-to-height ratio; TG: triglyceride LDL-c: LDL-colesterol; HDL-cholesterol; BF%: body fat; BMI: body mass index.

^a^p<0.05; ^b^p<0.001.

**Supplementary Table 2:** Cardiometabolic risk markers according to genotype in children aged 4 to 7 years. Viçosa, Minas Gerais, Brazil.

| Cardiometabolic Risk Markers | *FTO* | | |  |
| --- | --- | --- | --- | --- |
|  | **TT**  Mean (SD) | **AT**  Mean (SD) | **AA**  Mean (SD) | *p* value |
| BMI | 15,57 (1,71) | 15,88 (2,14) | 15,70 (1,71) | 0,552 |
| BF (%) | 17,21 (6,36) | 17,94 (7,83) | 18,01 (6,60) | 0,761 |
| TyG | 4,23 (0,21) | 4,27 (0,20) | 4,27 (0,18) | 0,287 |
| MAP | 71,11 (7,08) | 71,90 (8,57) | 72,40 (7,87) | 0,657 |
| WHR | 0,47 (0,03) | 0,47 (0,05) | 0,48 (0,04) | 0,465 |
| TG | 62,87 (26,07) | 67,72 (28,66) | 67,32 (25,03) | 0,465 |
| LDL | 98,65 (19,72) | 97,79 (23,27) | 101,32 (24,36) | 0,688 |
| HDL | 51,96 (11,36) | 49,73 (11,69) | 48,82 (10,19) | 0,264 |
| Cholesterol (non-HDL) | 111,37 (20,78) | 111,24 (24,59) | 114,82 (33,65) | 0,686 |
| Total cholesterol | 163,32 (22,35) | 160,98 (25,04) | 163,64 (35,40) | 0,765 |
| Blood glucose | 81,19 (7,08) | 82,46 (6,01) | 81,92 (6,33) | 0,370 |
| *No. of risk markers* |  |  |  |  |
| 0 | 5 (29,4%) | 10 (58,8%) | 2 (11,8%) | 0,496 |
| 1 | 19 (29,7%) | 34 (53,1%) | 11 (17,2%) |  |
| 2 | 27 (29,3%) | 46 (50,0%) | 19 (20,7%) |  |
| 3 or more | 20 (27,0%) | 37 (50,0%) | 17 (23,0%) |  |

TT: no polymorphism; AT: presence of a risk allele; AA: presence of polymorphism

BMI: body mass index; BF%: body fat; TyG: triglyceride-glucose index; MAP: mean arterial pressure; WHR: waist-to-height ratio; TG: triglyceride.

**Supplementary Table 3**. Association between Traditional and Snack dietary patterns (explanatory variables) and cardiometabolic risk factors (dependent variables) in children according to FTO genotype. Viçosa, Minas Gerais, Brazil, 2015-2016.

|  | Traditional | | | Snack | | |
| --- | --- | --- | --- | --- | --- | --- |
| Variables | TT  β (CI95%) | AT  β (CI95%) | AA  β (CI95%) | TT  β (CI95%) | AT  β (CI95%) | AA  β (CI95%) |
| TyG † | -0,01 (-0,06 - 0,05) | 0,0 (-0,04 - 0,04) | -0,01 (-0,06 - 0,05) | 0,0 (-0,05 - 0,05) | 0,02 (-0,02 - 0,06) | 0,02 (-0,03 - 0,07) |
| M AP† | 0,48 (-1,43 - 2,39) | -0,81 (-2,30 - 0,67) | -1,03 (-3,01 - 0,95) | -0,24 (-1,94 - 1,45) | 0,29 (-1,24 - 1,82) | 0,71 (-1,14 - 2,56) |
| WHR ‡ | 0,01 (-0,01 - 0,01) | 0,01 (0,0 - 0,01) | 0,0 (-0,01 - 0,01) | 0,01 (-0,01 - 0,01) | 0,0 (0,0 - 0,01) | 0,01 (-0,01 - 0,01) |
| TG † | -0,85 (-7,24 - 5,55) | -1,27 (-6,62 - 4,08) | -0,91 (-8,80 - 6,98) | -1,46 (-7,15 - 4,23) | 2,98 (-2,47 - 8,42) | 3,18 (-4,10 - 10,46) |
| LDL † | -0,18 (-5,50 - 5,14) | 0,41 (-3,96 - 4,79) | -2,15 (-12,27 - 7,98) | 2,08 (-2,63 - 6,79) | 0,65 (-3,81 - 5,11) | -1,80 (-11,22 - 7,62) |
| HDL † | -1,53 (-4,50 - 1,44) | 1,62 (-0,53 - 3,77) | 0,71 (-2,29 - 3,71) | 0,63 (-2,04 - 3,29) | 0,62 (-1,60 - 2,83) | -0,62 (-3,41 - 2,17) |
| Cholesterol (non- HDL)† | -0,01 (-5,64 - 5,44) | 0,30 (-4,34 - 4,93) | -2,39 (-12,99 - 8,22) | 1,61 (-3,30 - 6,53) | 1,38 (-3,34 -6,11) | -1,23 (-11,12 - 8,66) |
| Total cholesterol † | -1,63 (-7,63 - 4,37) | 1,97 (-2,81 - 6,64) | -1,68 (-12,57 - 9,21) | 2,24 (-3,08 - 7,57) | 2,00 (-2,83 - 6,83) | -1,85 (-11,97 - 8,28) |
| Blood glucose † | -0,09 (-1,93 - 1,76) | -0,20 (-1,27 - 0,86) | -0,85 (-2,63 - 0,92) | 1,03 (-0,58 - 2,64) | -0,12 (-1,21 - 0,97) | -0,79 (-2,44 - 0,86) |

95%CI: confidence interval (95%); TyG: triglyceride-glucose index; MAP: mean arterial pressure; WHR: waist-to-height ratio; TG: triglyceride; LDL-c: LDL-cholesterol; HDL-c: HDL-cholesterol;

TT: no polymorphism; AT: presence of a risk allele; AA: presence of polymorphism

†Adjusted for age, sex, fat percentage, screen time and maternal education;

‡ Adjusted for age, gender, screen time and maternal education.
